# Patients’ and professionals’ views related to ethical issues in precision medicine: a mixed research synthesis

**DOI:** 10.1186/s12910-021-00682-8

**Published:** 2021-08-31

**Authors:** Anke Erdmann, Christoph Rehmann-Sutter, Claudia Bozzaro

**Affiliations:** 1 Institute for Experimental Medicine, Medical Ethics Working Group, Kiel University (CAU), Kiel, Germany; 2 Institute for Medicine History and Science Research, University of Lübeck, Lübeck, Germany

**Keywords:** Precision medicine, Personalized medicine, Genomic medicine, Benefits, Access, Knowledge, Informed consent, Discrimination, Stigmatization, Data security

## Abstract

**Background:**

Precision medicine development is driven by the possibilities of next generation sequencing, information technology and artificial intelligence and thus, raises a number of ethical questions. Empirical studies have investigated such issues from the perspectives of health care professionals, researchers and patients. We synthesize the results from these studies in this review.

**Methods:**

We used a systematic strategy to search, screen and assess the literature for eligibility related to our research question. The initial search for empirical studies in five data bases provided 665 different records and we selected 92 of these publications for inclusion in this review. Data were extracted in a spreadsheet and categorized into different topics representing the views on ethical issues in precision medicine.

**Results:**

Many patients and professionals expect high benefits from precision medicine and have a positive attitude towards it. However, patients and professionals also perceive some risks. Commonly perceived risks include: lack of evidence for accuracy of tests and efficacy of treatments; limited knowledge of patients, which makes informed consent more difficult; possible unavailability of access to precision medicine for underprivileged people and ethnic minorities; misuse of data by insurance companies and employers, potential of racial stigmatization due to genetic information; unwanted communication of incidental findings; changes in doctor-patient-relationship through focusing on data; and the problem that patients could feel under pressure to optimize their health.

**Conclusions:**

National legislation and guidelines already minimize many risks associated with precision medicine. However, from our perspective some problems require more attention. Should hopes for precision medicine’s benefits be fulfilled, then the ethical principle of justice would require an unlimited access to precision medicine for all people. The potential for autonomous patients’ decisions must be greatly enhanced by improvements in patient education. Harm from test results must be avoided in any case by the highest possible data security level and communication guidelines. Changes in the doctor-patient relationship and the impact of precision medicine on the quality of life should be further investigated. Additionally, the cost-effectiveness of precision medicine should be further examined, in order to avoid malinvestment.

## Background

Precision medicine (PM) is a relatively new approach to individualize the prevention, diagnosis and treatment of various diseases. Since many diseases are caused by the interaction of genetic, lifestyle and environmental factors, and the outcome of treatments can also depend on genetic traits, the goal of an individualized therapy requires extensive data collection on the patient's genetic characteristics, lifestyle and environmental factors. Such an extensive data collection has become possible through digitization and the development of whole genome sequencing.

There are promising applications of PM, in particular for oncology, pharmacogenomics and various hereditary diseases [1]. However, PM is under development for many conditions. Since the authors are involved in a research consortium focused on the application of PM in chronic inflammatory diseases, we explain in the following the complexity of PM using the example of inflammatory bowel diseases (IBD).

Crohn's disease and ulcerative colitis appear to be the result of an interaction of the genome, exposome, microbiome and immunome. Genome-wide association studies have identified over 240 IBD susceptibility loci that potentially increase risk of disease. The studies present early life events, air pollution, smoking and diet as lifestyle and environmental factors. In addition, a reduced microbial diversity and a dysregulated immune response in the gut are also considered to be crucial factors in IBD development and progression [2].

A large number of treatment options are available for patients with IBD. PM pursues the goal of administering the right therapy to the right patient at the right time, while increasing the therapy response and reducing possible side effects. The reduction of side effects is particularly important in the therapy with thiopurines, as a NUDT15 gene variant can increase the risk of myelosuppression. The risk of pancreatitis as a result of treatment with thiopurines has also been described for certain genetic traits. To minimize the risk of side effects, therapeutic drug monitoring with biomarkers is required to optimize the drug dose [2].

Although some biomarkers are already available, the aim of future research is to develop further biomarkers in order to enable a personalized therapy based on "multi-omics data” [3] in IBD. This requires sharing data between research groups and the use of electronic health records (EHRs). To make sense of multiomics data, machine learning and algorithms will be necessary [2].

It is important to note that as precision medicine has evolved, other terms for this medical approach have emerged, such as *personalized medicine*, *genomic medicine, systems medicine* or *individualized medicine*. These terms emphasize different aspects: While the term *genomic medicine* emphasizes the use of gene sequencing technology, the term *precision medicine* fosters the, possibly unrealistic, expectation of a perfect fit to a patient outcome. In contrast, *personalized medicine* brings to the forefront the recognition that patients are more than their genes and interactions with the environment. They are influenced by their experiences, culture, education, and myriad other factors [4]. The concept of *individualized medicine* is quite similar to the concept of personalized medicine, as both emphasize the individual person. The term *systems medicine*, though, was derived from the theoretical concepts of systems biology and systems pharmacology and integrates these concepts into medical research and practice. Systems medicine accentuates the intensive collaboration between clinicians, biologists, pharmacologists, bioinformaticians and mathematicians, in which multidimensional sources of information are processed by computer modeling. Since this is also the case with precision medicine and personalized medicine, it seems that these concepts cannot be separated quite sharply. In fact, the terms precision medicine and personalized medicine have gained more popularity than the term systems medicine since 2000, as a simple search of the terms in the Pubmed database shows. The same analysis also reveals that the term precision medicine is used even more frequently than the term personalized medicine [5], which prompts us to mainly use the term precision medicine (PM) in this article.

### Ethical issues in precision medicine

Many ethical challenges regarding PM have already been reported. In addition to ethical issues concerning the massive data storage and data sharing, these challenges include:

- a possible discrimination by insurance companies and employers [6]
- discrimination in access to PM [6, 7]
- incidental findings in genetic testing [8]
- the lack of health literacy or “genetic literacy” for obtaining informed consent [9]
- the lack of scientific evidence of the efficacy and tolerability of treatments [10]
- the possibility of changing the patient-physician relationship by focusing on data [11, 12]
- and the increasing expectation on patients to contribute with data, time, effort and self-care [7].

Ethical issues in PM can be considered in light of various ethical theories. For our review, we choose Beachamps and Childress' common-sense based four-principles approach as a framework for our work, which includes beneficence, nonmaleficence, autonomy and justice [13]. These principles can be traced back to multiple ethical theories, e.g. the principle of autonomy to Mill’s Utilitarianism or to Kant’s Deontology, the principle of justice, for example, to the theories of Rawls [13]. But also other philosophers like Sen [14] or Nussbaum [15] have worked on justice. The principles of beneficence and nonmaleficence, both already contained in the Hippocratic Oath, have guided medical practice since antiquity [16]. Although respect for autonomy in multi-ethnic societies faces some challenges, as cultural, traditional or religious norms limit the autonomy of several groups [17], the principle of autonomy is also contained in the concept and declarations of human rights, which have been recognized by most nations. Here, the right to freedom is granted to all human beings [18] and the recognition of freedom is also the basis of the principle of autonomy in medicine [16]. In personalized medicine, the patient’s autonomy [19], justice [20] and nonmaleficence [21] provide a common framework for ethical reflection.

### Objective and research question of this review

Besides the theoretical discourse in the literature, many empirical studies have examined how patients and professionals perceive PM and which expectations, concerns, values and attitudes related to PM they have. These perspectives are quite relevant to the solution of ethical issues in PM, as they establish context sensitivity for the ethicist. Since morality is realized in social practices, empirical studies illuminate the moral experience of those involved in that practice [22]. For the ethical discussion of PM, empirical studies of the attitudes, expectations and perspectives of patients and professionals can provide a starting point that would enrich ethical reflection as these studies include moral beliefs, intuitions and reasonings. For this reason, a review of existing empirical studies representing the perceptions of professionals and patients in the field of PM seems useful to researchers and practitioners.

Empirical investigations on this topic have often been conducted in specific medical fields, mainly in oncology. However, to date, there has not been a review that analyzes and synthesizes the results from studies in different medical fields with regard to ethical issues. Our mixed research synthesis is intended to close this gap and deepen the understanding of patients’ and professionals’ views on PM. We believe that the experience gained in various medical fields can provide important information for the further, ethically reflected development of PM. Among professionals, we are particularly interested in the views of those directly involved in patients’ care. This group has a significant impact on ethically relevant issues, such as access to PM, communication about test results, information about treatment options and participation in research. However, we are also interested in researchers’ viewpoint(s), as researchers might consider the risks of data security and machine learning. Our review also serves to prepare an empirical research project on PM in chronic inflammatory diseases. In this project we intend to study the views of patients and professionals more precisely. By reviewing the literature available to date, we will answer the following research question: What are patients’ and professionals’ expectations, concerns, values and attitudes related to PM, including their understandings of risks and benefits?

## Methods

Because both quantitative and qualitative studies, as well as studies using a mixed method research design, are available to review the perspectives, views, or attitudes of patients and professionals regarding PM, we have chosen an integrated design of Mixed Research Synthesis as the methodology for our literature review. Mixed Research Synthesis integrates both quantitative and qualitative studies by transforming findings to combine them in one synthesis. Transformation can be performed in two different ways: (1) Qualitizing of findings means that quantitative findings are converted into qualitative form in order to combine them with other qualitative data; (2) Quantitizing of findings means converting qualitative findings in a quantitative form in order to combine them with quantitative data [23]. In this synthesis we mainly used the qualitizing approach since our epistemological interest is focused on the variance of perspectives, views or attitudes and respective justifications for certain positions of the interviewed persons. Results from quantitative studies were either presented with the numerical data from the original studies, or combined with findings from qualitative studies to form a meaningful and accurate statement for the thematic synthesis. By combining quantitative and qualitative data in this way, quantitative data can give more significance to qualitative findings and qualitative data can extend quantitative results.

To achieve the objectives of our review we defined inclusion and exclusion criteria for publications and designed our search strategy as follows:

### Inclusion criteria

- Content-related criteria
  Publications dealing with patients’ and professionals’ expectations, concerns, values and attitudes to PM, including their understanding of risks and benefits
  The above-mentioned publications must be relevant to the ethical discourse on PM
  Patients are limited to people with diseases or disabilities and representatives of patient organizations, who are (potential) users of PM interventions
  Professionals are limited to people who develop and implement PM interventions. These are physicians and other health care professionals (HCPs) in the clinical and outpatient context and researchers.

(b) Methods
  Empirical studies with qualitative and quantitative methods, reviews

(c) Information sources
  Journal articles, books, electronic databases

(d) Languages
  German and English

(e) Year of publication
  No limits

(f) Origin of publications
  Worldwide

### Exclusion criteria

- Content-related criteria
  Articles in which the views, expectations, perceptions, values, attitudes or concerns of patients or professionals to PM are not in focus (e.g., clinical trials)
  Evaluation studies of new PM curricula or learning models, e.g., for medical students, genetic counsellors

(b) Methods
  Articles in which the views of patients or professionals were not empirically investigated with scientific methods, but the authors merely presented their personal view or standpoint (commentaries, editorials, letters to the editor, normative analyses, journalistic individual interviews with experts, case studies, study protocols). Our intention was to include empirical studies that (1) were conducted with scientific methods and (2) went beyond providing the opinion of a single person, since we attribute a higher significance to such studies.

(c) Information sources
  Newspaper or magazine articles with journalists as authors or without any author.

(d) Population
  Users of direct-to-consumer genetic tests, economists, citizens, relatives, students, education providers, legal experts, representatives from regulatory authorities, reimbursement institutions, pharmaceutical industry, payer institutes, funding institutions, scientific associations, government officials, informatics, non-government-organizations, business experts and imprecisely identified stakeholders who do not meet the inclusion criteria explicitly.

### Information sources and search strategy

An initial search in five databases with the search terms *expectation, concern, value, attitude, risk, benefit, view* and *perspective* in combination with *patient, physician, doctor, stakeholder, expert, researcher* and *precision medicine, personalized medicine *or* genomic medicine* limited to title resulted in relatively few records. For this reason, we tried to broaden the search by using different types of studies as search terms and combined them with *precision medicine, personalized medicine* and *genomic medicine* in the title. This search strategy resulted in 1004 records (Table 1).

**Table 1:** Search strategy

Databases	Search term	Records
PubMed	((((((((quantitative study) OR qualitative study) OR participant observation) OR focus group) OR interview) OR survey) OR questionnaire)) AND ((((precision medicine[Title]) OR personalised medicine[Title]) OR personalized medicine[Title]) OR genomic medicine[Title])) Sort by: Best Match	458
Web of science	#1 (TI = precision medicine) #2 (TI = personalised medicine) #3 (TI = personalized medicine) #4 (TI = genomic medicine) #5 #1 OR #2 OR #3 OR #4 #6 (ALL = quantitative study) #7 (ALL = qualitative study) #8 (ALL = participant observation) #9 (ALL = focus group) #10 (ALL = interview) #11 (ALL = survey) #12 (ALL = questionnaire) #13 #6 OR #7 OR #8 OR #9 OR #10 OR #11 OR #12 #14 (#6 OR #7 OR #8 OR #9 OR #10 OR #11 OR 12) AND (#1 OR #2 OR #3 OR #4)	414
CINAHL	S1 TI precision medicine OR TI personalised medicine OR TI personalized medicine OR TI genomic medicine S2 TX quantitative study OR TX qualitative study OR TX participant observation OR TX focus group OR TX interview OR TX survey OR TX questionnaire S3 S1 AND S2	116
BELIT	(title:(precision AND medicine) OR title:(personalised AND medicine) OR title:(personalized AND medicine) OR title:(genomic AND medicine)) AND (all:(quantitative AND study) OR all:(qualitative AND study) OR all:(participant AND observation) OR all:(focus AND group) OR all:(interview) OR all:(survey) OR all:(questionnaire)) AND lang:(GER ENG) AND type:(book article)	12
Philosopher’s index	S1 TI precision medicine OR TI personalised medicine OR TI personalized medicine OR TI genomic medicine S2 TX quantitative study OR TX qualitative study OR TX participant observation OR TX focus group OR TX interview OR TX survey OR TX questionnaire S3 S1 AND S2	4
Total		1004

### Screening and eligibility assessment

**Fig. 1:**
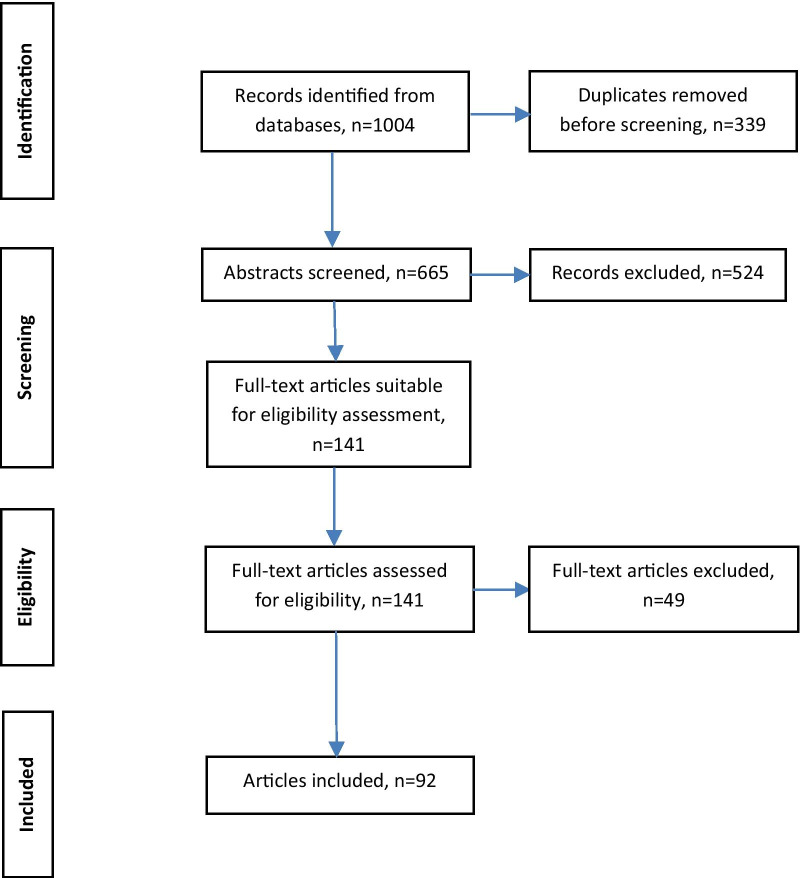
Review process

One researcher screened the abstracts and assessed the full-texts for eligibility. First, the number of records was reduced from 1004 to 665 by removing duplicates. The abstract screening process resulted in 524 titles being excluded as irrelevant, with 141 articles remaining for full-text-screening. During full-text-screening we excluded 49 publications due to the content of the publication, the method, the population studied or the source of information as defined in our exclusion criteria. In some publications a very heterogeneous sample was examined: patients, health care professionals, but also representatives of other groups that did not meet our inclusion criteria, such as representatives of health insurance companies. In such cases, the publication was only included if a sufficient subgroup analysis allowed a separate evaluation of the data. The whole article selection process resulted in 92 publications for this review. Figure 1 visualizes the review process:

### Data extraction

We listed the 92 publications in a data extraction spreadsheet and analyzed the data by identifying themes which related to our research question. During the analysis of professionals and patients’ expectations, perspectives, concerns, values and attitudes, topics that appeared to be irrelevant to the ethics of precision medicine also emerged. These topics included: the concept of PM, compatibility with personal values or professional beliefs, facilitators and needs, interprofessional communication or the changing role of scientists. Since our interest is only in ethically relevant themes, the final decision on whether to include a topic was based on the criterion of its relation to Beachamps and Childress' principles of biomedical ethics which are: beneficence, nonmaleficence, autonomy and justice [13]. We extracted relevant text passages from the publications and assigned them to the different themes. Finally, we summarized and discussed the identified themes iteratively.

## Results

While 62 studies investigated professionals’ perceptions on PM, 45 publications reported on the views of patients. Among the HCPs, many oncologists [24–32] participated in the included studies, but also other medical specialists are represented: nephrologists [33, 34], cardiologists [27], infectiologists [24], psychiatrists and clinical psychologists [35], pathologists [31, 32], gastroenterology specialty trainees [36, 37], geneticists and genetic counsellors [31, 32, 38–42], laboratory medicine professionals [43], pharmacists [44, 45], critical care intensivists [38], physician assistants [46] and nurses [30, 38, 46–48]. From the outpatient sector, primary care providers [46, 49–51] or family medicine providers [27, 52] participated in some studies. In many studies, researchers [42, 47, 53] were also represented. Those mentioned explicitly were clinical researchers [54–56], bioinformaticians [31, 32], laboratory scientists [31], experts from genome research [32, 57] and, in general terms, representatives from basic [54, 58] and translational research [58].

Since numerous studies have been conducted in oncology, the experiences and views of oncology patients are often found in studies selected for inclusion in our review [26, 56, 59–70]. However, studies we include also focused on patients with other diseases or disabilities. These were patients with chronic inflammatory diseases [71], chronic kidney disease [72], patients without a diagnosis, but with conditions presumed to be genetic (“diagnostic odyssey”) [64], patients with a chronic condition such as diabetes mellitus, hypercholesterolemia or hypertension [73], drug users (heroin, crack, cannabis) [74], patients with rare diseases [75] and people with disabilities [76–78]. Some studies did not provide any information about the condition of the patients included in the study.

**Fig. 2:**
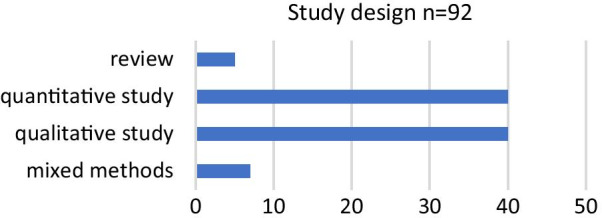
Methodology of included studies

Regarding the methodology of the studies, a quantitative study design was chosen as frequently as a qualitative design (Fig. 2).

We identified 13 topics related to the principles of biomedical ethics [13]. An overview of the association between the topics and the principles is shown in Table 2. Although some topics can be assigned to several principles, only the most obvious assignment was given here.

**Table 2:** Association between the topics identified in the publications and the principles of biomedical ethics

Principles [13]	Topics
Beneficence	Benefits of precision medicine, e.g., risk assessment, targeted therapies
Nonmaleficence	Professionals’ knowledge and competence Harm from test results or the testing process Lack of evidence Doctor-patient relationship Patients’ data provision and (personal) health-related work
Autonomy	Patients’ understanding and knowledge Communication and informed consent Privacy and confidentiality
Justice	Access to precision medicine Discrimination and stigmatization Profiteering with patient s’ data/biospecimens Health care costs

### Benefits of precision medicine

Various studies reveal that the majority of professionals have a positive attitude towards PM in general or towards interventions, which are mentioned under the label of PM [27, 34, 43, 45, 47, 50, 79–83]. In particular, the benefits of PM mentioned in the publications by professionals can be summarized as follows (Table 3).

**Table 3:** Benefits of PM from the perspective of professionals

Care process	PM has the potential to	Publication
Prevention	Be helpful for risk assessment, identifying a genetic predisposition or the prevention of disease	[27, 39, 47, 84]
	Supplement a family history	[50]
Diagnosis	Be helpful in diagnosing disorders	[43, 47, 49, 50, 85]
Decision-making	Increase therapy options	[31]
	Be helpful for the explanation of risks and therapy options	[33]
	Facilitate an informed decision for treatments	[34, 86]
Treatment	Tailor treatments and thereby make them more effective	[35, 50, 52, 84, 87–89]
	Reduce treatment side effects	[47, 79, 88]
Individual health outcomes	Improve patient outcomes	[27, 58, 79, 85]
	Improve quality of life	[47]
	Improve life expectancy	[31, 47]
Public health outcomes	Result in higher quality health-care	[58]
	Avoid overtreatment or wasting resources	[79]
	Reduce health care costs	[47, 58]
	Reduce hospitalization days	[88]
	Be favorable for the importance of medical progress	[82]

Apart from these positive assessments, studies also demonstrate that some professionals are uncertain about the value of genetic testing [51, 90] and doubt that all patients will benefit significantly from PM [30, 31, 42, 49, 54, 57]. In addition, Kichko et al. found in their study differences in attitudes between physicians from Pennsylvania and Bavaria. The Bavarian physicians were less convinced of the effectiveness of personalized drugs and less confident that personalized drugs had fewer side effects. They were also more skeptical than their American colleagues that PM can reduce hospitalization days or health care costs. The authors explained these differences with different health care systems, a different culture and history [88].

According to the studies, most patients hold positive, hope-filled views of PM. The willingness and interest to undergo PM interventions and a positive attitude towards them is high among the majority of patients surveyed [29, 63, 65, 69, 73, 74, 77, 91–93]. In the same way as the professionals, patients also combine PM with the possibility to tailor treatments or make them more effective [51, 63, 67, 68, 71, 94], avoid unsuccessful attempts of treatment [95, 96], improve drug prescribing [73] and reduce side effects [73, 95]. From the patient perspective, PM has the potential to minimize disease impact [68], improve quality of life [71] and decrease chronic pain for patients with chronic inflammatory diseases [71]. By providing information about their condition and genes, PM can empower patients to “self-advocate” [64], especially when they have to make informed decisions [79]. Remarkable is the relevance that patients attach to PM in terms of prevention [51, 94, 97]. Genomic risk knowledge gives patients the opportunity to change their lifestyle for the purpose of health improvement, and in this way, they gain more control over their own health [61]. Additional benefits mentioned in this context are the possibility to learn about ancestry, help family members [51] and improve family planning [97]. Some patients hope that new treatments will be discovered [97], that future patients will benefit from research, and medical care in general will be improved [98].

### Patients’ understanding and knowledge

Although many patients are convinced of the benefits of PM, their actual knowledge of its potential appears to be limited. Many professionals reported in the studies that patients have little or no awareness about the concept and potentials of PM [25, 58] and these professionals are skeptical about whether patients have the ability to understand PM [47, 56, 96]. One reason for this could be that the terms "stratified," "precision" or "personalized" medicine are rarely used in medical consultations [59] and in discussions within patient organizations [96]. Some physicians tend to simplify the complex issues in their conversations with patients and use other terms like “evidence-based medicine” [56]. However, it seems that physicians sometimes underestimate patients’ knowledge. For example, Ciardiello et al. did not always show agreement between doctors and patients in assessment of patient knowledge. While 85% of patients felt well informed about their treatment after the explanation of the doctor, only 23% of doctors agreed that their patients were well informed [29].

Conversely, some professionals perceive patients as the "drivers" [49] of PM, especially when it comes to the availability of genetic testing [49]. Informed by the media [26, 73], especially patients in oncology ask their physicians about PM more frequently than patients with other diseases [27]. For example, Tejpar et al. showed that the majority of patients in oncology are aware of genetic testing to determine which cancer treatment might be the best for an individual person [70]. However, as other studies have demonstrated, many patients generally have a limited knowledge about PM [65, 71, 99–102], genetic testing [62, 69, 95] and pharmacogenomics [73, 101] and also express difficulties in understanding [51]. Patients seem to be more familiar with older terms like “gene” or “DNA” than with newer ones like “pharmacogenomics” or “biobank” [101]. Even if patients are aware of the phrase “personalized medicine,” 19% of them do not have the right idea of what PM really is and combine PM, for example, with a constant doctor-patient contact or with the participation of patients in medical decision-making [63]. In addition, representatives from patient organizations note that patients often have difficulties in understanding basic medical information and that patient education will be a major task for patient organizations [96]. Hellwig et al. pointed out that patients’ understanding is also in the interest of health care providers, as it facilitates the communication process [86].

### Professionals’ knowledge and competence

Professionals also describe a lack in their own knowledge or a limited understanding, as quantitative studies demonstrate [36, 37, 44, 81, 82, 92, 103, 104]. For example, in a study with 100 UK gastroenterology trainees, most of them believed that their training had not prepared them for practicing PM [36, 37]. Carroll et al. surveyed 361 family physicians in Canada about their knowledge in genomic medicine and found a median knowledge score of 6 (on a scale from 0 to 10) with a wide range from 0 to 10. The self-reported level of confidence in practicing genomic medicine tasks was low in that study [85]. Alharbi et al. assessed the knowledge of 126 South Arabian physicians about PM in diabetes mellitus management and found that 82.5% of the participants had poor knowledge in this area [82]. These and other studies [37, 44–47, 58, 86, 105–108] reveal the need for further education of many health care providers, however, this requirement may vary between professionals in different fields [53, 81]. An exception seems to be the skills of physicians in oncology. A comparative study with oncologists, cardiologists and family doctors concluded that oncologists felt better informed, were more able to interpret test results and more confident in discussing the results with their patients [27]. Nevertheless, even among oncologists there seems to be some uncertainty at times regarding the choice of the right treatment [28, 31] and the interpretation [30–32] or explanation [31] of genomic data. Another exception of better knowledge was also demonstrated among clinical geneticists and genetic counsellors. As the study by Nisselle et al. highlighted, the majority of genetic counsellors (74.2%, n = 271) and clinical geneticists (87.0%, n = 83) had attended continuing professional development in genomics during the two previous years [41].

### Access to precision medicine

Due to the high demand for research, resources and infrastructure required for PM, it is questionable whether access to it will be open for all patients in the world. For example, in Saudi Arabia, where healthcare is predominantly taxpayer-funded [109], 27.8% of physicians surveyed (n = 126) doubted that patients would have easy access to PM [82]. In Korea, a state where the percentage of out-of-pocket-payments in healthcare is 35% [110], most of the health care professionals surveyed believed that only a few patients would have access to PM and 84.8% of the participants (n = 542) were concerned that this would increase disparity in public health [47]. In Europe as well, where healthcare is primarily funded through government-regulated public health insurance systems, taxpayers and private insurance policies [111, 112], PM is considered by researchers, healthcare providers and patient representatives to have limited availability. This is because only a few programs are already in place. [58]. In the US, where about 50% of healthcare funding is private [111, 112], 78% of patients, HCPs and patient representatives surveyed (n = 72) expressed concern about the difficulty for patient advocates to help patients gain PM access [68].

One factor that could limit access to PM might be the lack of coverage by health insurance companies for medication [54], tests [29, 80] or genetic counselling [90] and the inability of patients to pay for PM out of pocket [73, 90, 97]. Physicians are described as gatekeepers and their decisions on medical appropriateness, which may vary and be determined by guidelines [29], condition access to PM interventions. Patients perceive these variations as disparity in access and some patients also suspect that physicians withhold access because of the high cost [26]. In addition, age [54, 79], the availability of tests [29, 80], the clinic or hospital location, patient attitudes, norms and education, as well as social factors like discouragement by significant others are identified as barriers for PM [90]. Another factor could be the lack of communication to the public about the options PM offers. This possibility was mentioned by a member of the National Black Nurses Association in the USA, who questioned why the All of Us program is only communicated in English and Spanish [48]. That access to PM could be a question of ethnicity was shown by the Petersen et al. study. Only 38.5% of doctors surveyed (n = 104) in the US believed that PM is available to all ethnic groups. [103]. Ratcliff et al. cited a study which shows that ethnic minorities are less likely to embrace PM technology. Ethnic minorities and individuals with lower socioeconomic status seem to be less aware of technologies and less likely to use them [113]. The findings from a focus group study from Kraft et al. (2018) drew attention to the inability of US immigrants to navigate the healthcare system which can result in a lack of trust in the healthcare system [114]. The US-American survey from Diaz et al. (2014) revealed that for non-Hispanic, Black respondents disparity in access due to the inability to pay for PM is of greater concern [97]. But access to PM for ethnic minorities could also be limited for another reason. According to patient representatives, the availability of PM for certain groups could also depend on the profitability for the pharmaceutical industry of providing appropriate medicines for people with certain genetic characteristics [96].

Table 4 summarizes the factors which could limit access to PM from the perspective of professionals and patients. While factors limiting the offer and provision of PM prevail on the professional side, patients see limitations in their own possibilities to access it.

**Table 4:** Factors which could limit access to PM from the perspective of professionals and patients

Factors from the perspective of	Professionals	Patients
Lack of coverage by health insurance companies	[29, 54, 80, 90]	
Inability to pay out of pocket	[90]	[73, 97]
Decisions of physicians on medical appropriateness	[29]	[26]
Not part of treatment guidelines	[29]	
Availability of tests	[29, 80]	
Decisions of physicians due to high costs	[29]	[26]
Age	[54, 79]	
Clinic or hospital location	[90]	
Patient attitudes, norms and education	[90]	
Discouragement by significant others	[90]	
Lack of communication to the public	[48]	
Belonging to a specific ethnic group	[103]	[113]
Lack of profitability for pharmaceutical industry		[96]

### Discrimination and stigmatization

In addition to possible disparities in access to PM, professionals and patients fear discrimination based on the results of genetic testing [27, 79, 92, 97], particularly by insurance companies or employers [46, 51, 54, 63, 86, 94–96, 98, 115]. The majority of physicians interviewed in the USA and Germany would therefore not grant access to genetic information to employers and health insurance companies [88]. However, an American longitudinal study of 823 patients shows that none of the respondents had problems with health insurance after one year and only three patients reported problems with life insurance or long-term care insurance [92].

But genetic information also opens up the possibility of stigmatization of certain groups [57]. In a qualitative study, an African American participant explained this more precisely: this participant feared being put into a certain racially determined category because of genetic information which is much more present in that specific ethnic group [98].

From the perspective of professionals an additional unacceptable use of data would be to refuse someone an organ transplantation because of their genetic predisposition to organ rejection [33, 34]. Additionally, health advocates in oncology have expressed concern that patients who are not suitable for personalized treatment will not receive appropriate support [68].

### Privacy and confidentiality

Some professionals consider apprehension about data confidentiality not being guaranteed [45, 88, 116]. According to some researchers, the greatest danger here is in the sloppiness of people who work with the data and load it, for example, onto their laptop or flash drive [116]. As an indication that data security cannot be guaranteed, some patients mention the hacking of financial or online data and emphasize the need for harsh penalties [98]. People with a drug addiction history are concerned that medical practitioners cannot refuse information requests from courts [74]. A survey of medical practitioners and patients in India gives a somewhat more optimistic impression. Some of the respondents believe that data confidentiality is secured by advances in technology, while others are suspicious of whether the person entrusted with confidentiality can guarantee data security [105]. However, it seems that the human factor is the greatest weakness of the system.

Apart from data security, one publication raises another problem: the confidentiality of genetic information to families rather than to individuals, which means that information about a genetic disposition is shared with all at-risk family members. This “familial approach to confidentiality” [117] is conceptualized in UK genetic guidelines. In the study from Dheensa et al., 80 HCPs were interviewed in focus groups about their arguments for or against this approach to confidentiality. One of the respondents' arguments was that a familial approach could affect family relationships and the patient's trust in the health care system. A second argument concerned their resources for sharing information and the fear that sharing would make them more vulnerable to liability issues [117].

### Harm from test results or the testing process

Although the benefits of genetic testing are not questioned by many patients and professionals, some agree that test results or the testing process itself can also cause harm. This harm can occur, for example, if patients or professionals misinterpret the results [90] and for this reason make the wrong therapeutic decision [32, 92]. Some studies indicate that women who have been tested for breast cancer predisposition have undergone preventive surgery, even though the result was considered to be uninformative by their physician [40, 92]. One professional reported that a patient committed suicide after receiving the diagnosis of Huntington disease on the phone [40]. Besides these adverse events, professionals and patients in some studies report psychological implications from knowing [31, 32, 46, 51, 63, 113] or while waiting for the test results [26]. Incidental findings [79, 96], variants of unknown significance [79] or results that indicate a high risk for an incurable disease can affect the well-being of patients [51]. In some studies, patients explicitly state that they do not want to know certain results, e.g., about a genetic predisposition to a disease [95], especially if it cannot be cured [61, 118]. Not knowing enables people to keep hope, a positive self-perception and remain optimistic [113]. From the perspective of professionals during tumor board meetings, McGraw et al. in 2017 showed the potential harm from patients receiving information about test results, and also the harm when test results are withheld: the omission of findings can result in a missed opportunity to learn about a serious disease. Withholding test results may be useful in the future, but is an expression of “excessive paternalism” [32]. Therefore, some authors have argued that the preferences of each patient on receiving their test results need to be identified and addressed [57] and that professionals have the responsibility to protect patients and families from harm [42].

Although many of the above-mentioned studies indicate that patients can imagine possible harm from test results or the testing process, the review by McFarland et al. presents a slightly different picture. It found no evidence that patients have any concerns about tumor testing for the purpose of targeted therapy. The authors attributed this difference to the fact that this kind of testing is not a test for an inherited cancer risk, but rather a targeted therapy, similar to chemotherapy [100]. For patients, therefore, it seems to make a difference whether it is a question of identifying a risk for an illness or of searching for the right therapy for a severe disease that already exists.

### Communication and informed consent

For various reasons, professionals perceive communication on PM research, tests and therapies and obtaining informed consent as being increasingly complex [31, 79]. For example, in cancer PM the complexity and ambiguity of the evidence seems to make it difficult to decide which test results should be communicated to patients [32]. Challenges in obtaining informed consent include complex discussions about risks, the length of documents, the lack of understanding by professionals and the resources required [42]. Several quantitative studies indicate that patients want to be fully informed about tests [71], the results [51, 71, 72, 78] and treatment options [29]. Further, they wish to be involved in the decision-making process [29, 71]. However, genetic tests arouse suspicion among members of ethnic minorities who fear not being fully informed about the purpose and further use of the tests. Therefore, some patients are reluctant to use targeted therapies [74].

According to the professionals, another reason for the growing complexity of the discussions is the increasing presence of PM in the media. Some doctors feel that this increased media attention means discussions and decisions on testing are becoming more complicated and that they sometimes feel forced to order a test [26]. Clinicians and researchers also criticize that the media raises unrealistic expectations among patients, which cannot always be fulfilled [31].

With regard to participation in research, patients from different ethnic groups express skepticism about the consent process and are suspicious about whether all consented rules are actually followed. Many would therefore prefer to have the right to withdraw their consent at any time of the study [98]. Some participants would like to have separate consent for biospecimens and EHR data, as they see a risk for misuse of DNA in future research [115]. The Edwards et al. survey reveals that the majority of patients wish re-consent, if their data are used for a different, but related or unrelated health condition. A re-consent should also be granted when a child reaches the age of majority. Despite these preferences, the majority of respondents believe that the benefit of broad consent outweighs the harm, highlighting the feasibility and relevance of research [60].

### Lack of evidence

For clinical practice, test results are only useful when they deliver reliable and actionable information that can be used for clinical decisions. However, interpreting multiomics, clinical and lifestyle data becomes complicated by inadequate validation of biomarkers and insufficient evidence of clinical utility, which leads to clinical uncertainty [58]. Many clinicians and researchers are well aware of the limited [27, 30, 31, 35, 42, 79, 84] or ambiguous evidence [32] for the meaning of test results and possible treatment outcomes and regard the lack of practice guidelines as a barrier for the implementation of tests [27, 79]. Some patients also doubt the accuracy of the tests [63, 73] or the value of the test results to influence their fate [61]. In summary, the problem of small samples in clinical trials caused by patient stratification leads to an awareness of unclear evidence among professionals and creates uncertainty about what conclusions can be drawn from the tests for therapeutic decisions.

### Doctor-patient-relationship

Some publications indicate that professionals expect a change in the doctor-patient relationship through PM. In the studies by Dion-Labrie et al., some of the physicians interviewed expressed concern that behind the objective data, the human aspects of the doctor-patient relationship and the view on the whole person could be lost. The gain in objectivity carries the risk that less room is given to feelings in communication with the patient. [33, 34]. Another study suggests that the availability of large amounts of data on the patient increases the knowledge lead of the doctor, with the risk that the doctor will use this knowledge to make paternalistic decisions [56]. From the patient perspective, inequalities in access to PM can put a negative strain on the doctor-patient relationship, for example, the patient knows about this option but their doctor has not offered it to them [26].

### Patients’ data provision and health-related work

The willingness to participate in research and to provide genetic information, lifestyle, environmental or medical data was examined in a Korean study with 526 participants. The majority of the clinicians, researchers and health professionals surveyed showed a clear willingness to participate, although this willingness is higher when it comes to their own treatment as opposed to that of others [47]. Among patients there is also a high readiness to participate in trials and donate data or biospecimens [62, 70, 72, 76, 77, 98, 99], but trust in professionals [98, 101], costs, receiving counseling about test results and privacy [101], as well as the donor’s religion and culture [98] seem to be important for the decision. Significantly fewer patients support the use of smartphone apps to track lifestyle, behavior or environmental influences [72, 76, 77]. Possible factors behind this reluctance could be that this type of self-monitoring continuously confronts patients with their illness and that they get tired by digital interactions. As a result, some patients evade health care provider expectations of recording data using smartphone apps or wearables [113]. In addition, several studies show that the large number of tests carried out in the course of PM is a considerable burden for patients [71], as it is associated with time spent waiting at a clinic [59] and possibly with the need to travel [61]. These burdens are factors that determine whether patients have PM tests or treatments performed [61, 67].

### Profiteering with patient s’ data/biospecimens

Although there is generally a high willingness among patients to donate data and biospecimens for precision medicine research, one study also suggests that patients expect a corresponding countervalue when pharmaceutical companies earn large sums of money from drug development that was made possible by patients' donations. In the study, patients refer to the case of Henrietta Lacks, in which her cells (taken from tumor biopsy) were cultured on 1951 without her knowledge/consent and resulted in the HeLa immortalized cell line that is still being used in research today. The Lacks case highlights the injustice that results from making an enormous profit from the biospecimens of unsuspecting patients. Some participants in the study argued that if biospecimens contributes to corporate profit, patients should be compensated [98, 115].

### Health care costs

In several studies, professionals and patients express concern about the high cost of PM. Although professionals expect a reduction of the overall health care costs in the long term [47], some health care professionals question the cost–benefit ratio of PM [30, 54, 84, 105]. Compared to the costs of chronic diseases, PM costs are perceived as not only being massive, but also caused by a much smaller proportion of the entire patient population [30]. It is therefore questionable whether other care interventions would not have a greater benefit and deserve better funding [119]. But PM costs are also problematic for other reasons. For example, professionals [45, 120], patients and representatives from patient organizations are concerned about whether health insurance companies will cover the costs [51, 63, 76] or patients can pay for them [61, 95, 96]. And indeed a study from Europe reports a certain reluctance on the part of health insurers to cover the costs for PM, as the evidence is insufficient and incentives too small [58]. The willingness of patients to pay for PM themselves is apparently higher when the purpose is to treat a serious illness, such as cancer [73]. Nevertheless, most American and German physicians agree that the costs for PM should not be covered by the patients themselves [88]. Some patients also worry that if a drug is not prescribed frequently enough, pharmaceutical companies will raise the price or stop its production [98]. This would make PM unavailable to small groups with certain traits, which could put these groups at a disadvantage compared to others.

## Discussion

### Implications of the findings in context of existing research

Our review results show that many professionals and most patients expect high benefits from PM and have a positive attitude towards it. However, there is more doubt among professionals as to whether patients actually benefit from PM, which may be related to the fact that there is less evidence for positive effects of PM than in conventional medicine. The high specificity and costs of therapies mean that drugs are tested in smaller clinical trials rather than in large randomized controlled trials and even if several small trials have shown no risks, a level of uncertainty remains [10]. It is quite understandable that HCPs, whose training has so far been oriented on the ideal of evidence-based medicine, have more doubts here. In addition, many HCPs do not feel sufficiently trained and are uncertain about the interpretation of genomic data and the choice of the right therapy. Although machine learning systems can support the interpretation of data and develop therapy recommendations, doctors should not blindly rely on a machine without checking its results. Training programs on medical informatics for physicians will therefore become necessary [121]. In the meantime, it seems to be useful that different specialists, e.g., physicians, bioinformaticians, geneticists and genetic counselors work together in an interdisciplinary network, as is already the case in some places. Bioinformaticians in particular are considered to be of high importance [47]. The complexity of PM also confronts health care professionals with the challenge of communicating with patients and obtaining informed consent. Since geneticists and genetic counsellors seem to be better trained in genomics [41], their involvement appears to be essential.

However, improving professional competence and interdisciplinary cooperation alone cannot overcome patients’ lack of knowledge and understanding of PM. Rather, patient education about PM must be provided in an understandable way to ensure that patients can make autonomous decisions. By taking the level of health and genetic literacy into account, patient education must also be personalized. The concerns of ethnic minorities, who seem to have a stronger distrust of genetic testing [74] and the needs of people with disabilities [76] should be taken into consideration. The text-based education practiced in many places cannot meet these requirements [56]. Here, new formats need to be found and implemented. A promising approach could be short video sequences like those used by some researchers in their research projects [98, 115]. Since 3.3 billion people by today own a smartphone [122], those videos could easily be downloaded and viewed with such devices. Here, too, patients’ digital competence must be considered. Today, not all patients, especially the elderly, are ready to meet the challenges of digitization or have the appropriate IT infrastructure. Transmission of videos in patient waiting rooms seems a possible alternative. However, these additional services should not replace a personal consultation with a professional, but only supplement it.

The results of the review reveal that many professionals doubt that all patients who would benefit will have access to PM. But if PM can actually fulfil the hopes for more targeted therapies, fewer side effects and an improved quality of life, then the ethical principle of justice requires access to PM for all people in need. The individual patient's insurance status, ethnicity, age or place of residence should not limit access to PM. This principle seems particularly difficult to realize both nationally and internationally, as many health care systems will not be able to cover the costs of a nationwide PM implementation, However, full cost coverage by health insurance companies would make access much easier. Since health care providers act as gatekeepers and control access to PM, the development of guidelines must keep pace with PM implementation. Physicians in the outpatient sector, patient organizations and the public should be kept continuously informed of current developments.

But even if everyone had access to PM—regardless of their personal characteristics—at this time, not everyone would benefit from it equally. The reason for this is a recruitment bias in PM, namely the fact that most genetic data are obtained from Northern Europeans. This means that for other population groups, there is an increased probability that the result of a genetic test will produce variants of unknown clinical significance. Thus, it is not known whether these variants can cause a disease, as they have not yet been researched. The result is that non-Northern Europeans benefit less from developments in PM [123]. In addition, social circumstances, such as having to travel long distances to a medical center, fear of sanctions for work absences or language and cultural barriers can also make access to PM difficult [7]. And if medical consultations can only be arranged online or even the meeting with the physician takes place via video consultation, people who do not have the digital competence or the appropriate equipment can thus be excluded from PM [7].

As our review reveals, the cost–benefit ratio of PM is not considered balanced by some professionals and the critical question is raised of whether other interventions promise greater benefits and deserve better funding. Rey-Lopez et al. in their 2018 study also criticized the exorbitant expenditure on PM and suggested instead that more resources should be invested in improving people’s living conditions and health-related behavior. For example, policies that counteract climate change, such as a meat-free diet, abandonment of motor vehicles in favor of more physical activity, would be equally or even more beneficial to health and reduce mortality. They argue that technology-based prevention programs like fitness apps for weight reduction, which are sometimes promoted by PM, have not had a better effect on health than conventional programs. Rather, the required behavioral changes could be achieved by addressing social, cultural, economic and environmental circumstances [124]. In addition to better prevention, there are probably many other areas of healthcare in some countries where more money needs to be invested. Considering the cost-effectiveness of PM, Kasztura et al. revealed in their scoping review that PM is to date at least as effective as conventional care. The authors conclude that the cost-effectiveness of PM should be further investigated with different research approaches [125].

Our findings show that patients and professionals are concerned that genetic information could end up in the hands of insurance companies and employers and thereby lead to “genetic discrimination” [126]. This concern is not unjustified. For use in healthcare, genetic information needs to be integrated into electronic health records and these are increasingly becoming a target for attacks by cybercriminals [127]. Furthermore, as one study reveals, human error represents an additional risk [116]. Data security must therefore be given high priority, but the collection of genetic, environmental or lifestyle data should also be limited to information that is really necessary. Before data collection, patients should be informed about the possibility of data breaches to enable patients to balance their personal benefits and risks.

As we have seen in the studies, the (unwanted) disclosure of test results can harm patients in many ways. Experiencing a potential risk of illness can be a considerable strain on people, especially if the illness is incurable. However, avoiding telling patients about their genetic disposition for a treatable disease can also be harmful, as it deprives patients and their families of the opportunity for prevention and screening [10]. Therefore, for each genetic test, the patient's preferences regarding the information about disease risks should be respected, as already contained in existing legislation in some nations [128]. In addition, incidental findings associated with genetic traits of unknown clinical significance or findings of misattributed parentage [8] are problematic, because communicating these findings to patients and their families can also trigger stress and affect family relationships. By obtaining informed consent, the information on possible incidental findings should be kept short and concise so that patients understand the complex issue and are not confused by the various options for decision [10]. The possibility of re-contacting the patient is seen as a moral obligation if findings of previously unknown clinical significance can be better understood in the future and linked to therapeutic options [9].

In merely two studies, professionals talked about the possibility of changes in the doctor-patient relationship. This concern is perhaps more likely to be expressed by ethicists. By restraining the focus of the physician on measurable parameters and bodily functions [119], the patient's personality, their history, values and ideas about life risk being lost [11]. Salari and Larijani’s work described the danger that the patient will be perceived by the physician only as "genetic material" [12]. The complete digitalization of human life with omics-based data could lead to alienation between physician and patient in which the individuality of the patient increasingly disappears behind the data and algorithms [129]. The question of whether such changes can actually be observed in practice should be the subject of further empirical research.

Our review shows that patients and professionals are highly willing to participate in clinical trials and donate data and biospecimens to PM. Nevertheless, some people would like to be compensated for their donation if pharmaceutical companies gain a high profit with it [98, 115]. The question of whether patients should receive something in return for their donation or “gift” of data and biospecimens is one Lee discussed in a 2020 study [20]. In this work, Lee referred to anthropologist Marcel Mauss and considered the gift in the context of social relationships, where a gift is inextricable from obligations and reciprocity. The metaphor of the gift requires something in return, and some authors see the return of individual genetic information as a way to honor the gift in precision medicine research. However, such an approach must take into account that the value of the genetic information (e.g., actionable, non-actionable) is understood by the research participants [20] and also in their interest. The wish not to know individual genetic information should be respected.

In contrast to biospecimen donations, patients’ willingness to donate is less evident for the use of their data obtained from smartphone apps that collect lifestyle, behavioral or environmental information. A possible reason for this could be that these apps put patients under increasing pressure to continuously optimize their health condition, for example through physical exercise or practicing a certain diet. Prainsack argued in 2017 that PM is not possible without patients contributing data, time, effort and self-care, and describes this contribution as "patient work" [7]. Although patients have been encouraged to adopt a healthy lifestyle for a long time, the knowledge of the importance of the exposome and modern possibilities of constant data monitoring are leading to patients having a stronger responsibility for their health or recovery. This responsibility transforms patients into what Zwart calls, “bio-citizens [who] are expected to measure and monitor their bodies and their everyday lifeworld in real time, continuously and automatically” [130]. As the use of smartphone apps and wearables will continue to increase, the possible psychological impact of continuous self-optimization needs to be further researched.

### Limitations of the study

Our study has the following limitations: First, our search strategy was limited to three terms in the title of the publications (precision medicine, personalized medicine and genomic medicine). These terms are all used synonymously for PM, but each emphasizes different aspects of PM. We omitted the terms *individualized medicine* and *systems medicine* to ensure the feasibility of the study in a limited time frame. Compared to the words we have used in our search, the term *individualized medicine* seemed to be too unspecific and the term *systems medicine* seemed to be less common [5]. The use of these search terms would certainly have resulted in an additional number of relevant studies. A second limitation relates to the screening and eligibility assessment, which could only be performed by one researcher. A second person would have increased the reliability of the eligibility assessment.

## Conclusions

### Main conclusion

National legislation and guidelines in many countries have already addressed and solved a number of problems associated with PM. However, from our perspective, some problems still require more attention. If we approve the four principles for biomedical ethics of Beauchamp and Childress as a basis for ethical decisions, which is common in the European and North American context, then respect for the autonomy of the individual must be interpreted in the contexts of PM and ensured in the first place. Autonomy in PM is realized primarily in free and informed decision-making, so respect for autonomy demands comprehensible education and support. Our results show, however, that patients have difficulties in understanding some of the underlying ideas of precision medicine, therefore an adaptation of information documents to make them more understandable and an improvement of patient education seems necessary. Respect for autonomy could be improved by taking into account individual health literacy when educating people about decisions to participate in tests, therapies, research or self-tracking. In more family or community-based societies where therapeutic decisions are not made on the basis of the patient's will alone, it would make sense to include the community in the development of PM educational activities, in order to learn how to improve the knowledge and understanding of those involved in decision making.

Should hopes for precision medicine’s benefits be fulfilled, then the ethical principle of justice would require an unlimited access to precision medicine for all people. As our results show, both patients and professionals, have considerable doubts about this. We agree with the view that justice is easier to realize in public health care systems, which are funded by taxpayers or by government-regulated public health insurance plans. The obvious reason is that in these health care systems access is not dependent on personal financial resources. However, it is questionable whether the cost–benefit ratio of precision medicine is advantageous and whether other patient groups that do not benefit from PM are not ultimately disadvantaged due to lack of financial resources. The cost-effectiveness of PM should therefore be further investigated.

The ethical principle of nonmaleficence requires that PM should in no case harm the patient. However, from the perspectives of professionals and patients, the collection of health data carries a high risk of misuse. Thus the principle of nonmaleficence requires data collection of genetic, environmental or lifestyle data to be limited to what is absolutely necessary. Data security must be given high priority.

The question of whether the physician–patient relationship is altered by the physician's focus on multi-omics data and whether the patient's subjective experience is thereby eclipsed also relates to the ethical principle of nonmaleficence. This too requires further investigation.

The most important question seems to be whether and under which conditions PM really has the potential to improve patients’ quality of life – the life they themselves would judge as “good” – or whether it is subjectively perceived as worse, due to an over-compliance with the imperative of self-optimization and the necessary constant work on one's own health. This question, which refers to the ethical principle of beneficence, also needs to be more closely explored in future research.

### Explanation of the importance and relevance of the study

This is the first review that presents and analyzes results from qualitative and quantitative studies regarding the perception of patients and professionals on ethical issues in PM.

## Data Availability

The dataset used and analyzed during this study is available from the corresponding author on reasonable request.

## Abbreviations

PM: Precision medicine
IBD: Inflammatory bowel disease
EHR: Electronic Health Record
HCP: Health Care Professional
